# Physiochemical and antioxidant properties of roselle-mango juice blends; effects of packaging material, storage temperature and time

**DOI:** 10.1002/fsn3.174

**Published:** 2015-01-27

**Authors:** Beatrice Mgaya-Kilima, Siv Fagertun Remberg, Bernard Elias Chove, Trude Wicklund

**Affiliations:** 1 Department of Chemistry, Biotechnology and Food Science, Norwegian University of Life SciencesP.O. Box 5003, 1432, Aas, Norway; 2 Faculty of Agriculture, Department of Food Science and Technology, Sokoine University of AgricultureP. O. Box 3006, Morogoro, Tanzania; 3 Department of Plant and Environmental Sciences, Norwegian University of Life SciencesP.O. Box 5003, 1432, Aas, Norway

**Keywords:** Juice blend, mango, physiochemical properties, roselle, storage conditions

## Abstract

A study was conducted to determine the effects of packaging materials, seasonality, storage temperature and time on physiochemical and antioxidant properties of roselle-mango juice blends. Roselle extract (20%, 40%, 60%, and 80%) was mixed with mango juice and stored in glass and plastic bottles at 4°C and 28°C. Total soluble solids, pH, titratable acidity, reducing sugar, color, vitamin C, total monomeric anthocyanins, total phenols, and antioxidant activity (FRAP) were evaluated in freshly prepared juice, and after, 2, 4, and 6 months of storage. The results showed that total soluble solids, reducing sugars, and pH increased with storage times under different storage time, irrespective of packaging materials. The acidity, color, total monomeric anthocyanin, vitamin C, total phenols, and antioxidant activity decreased during storage irrespective of storage temperature and packaging material. Loss of anthocyanins, total phenols, and vitamin C content were higher in blends stored at 28°C than 4°C.

## Introduction

*Hibiscus sabdariffa* L. (family *Malvaceae*), commonly known as roselle, red sorrel, or Karkadè, is widely grown in Africa, South East Asia, and some tropical countries of America (Abou-Arab et al. 2011; Sagayo-Ayerdi et al. 2007; Amor and Allaf 2009; Cisse et al. 2011*)* Roselle produces red edible calyces with unique brilliant red color and flavor. The calyces are commonly used in the production of jelly, juice, jam, wine, syrup, gelatine, pudding, cake, ice cream, and flavoring (Tsai et al. 2002; Tsai and Huang 2004; Duangmal et al. 2008 and Hussein et al. 2010). The beverages produced by *Hibiscus sabdariffa* calyces are called hibiscus tea, bissap, roselle, red sorrel, agua de Jamaica, Lo-Shen, Sudan tea, or karkade (McKay et al. 2010).

Two anthocyanins are dominant in roselle calyxes, delphinidin-3-sambubioside, also known as delphinidin-3-xylosylglucoside or hibiscin, and cyanidin-3-sambubioside, also known as cyanidin-3-xylosylglucoside or gossypicyanin. In addition, two minor anthocyanins, delphinidin-3-glucoside, and cyanidin-3-glucoside are present (Wong et al. 2002; Amor and Allaf 2009; Cisse et al. 2011). Roselle anthocyanins render health benefits as a good source of antioxidants as well as a natural food colorant (Tsai et al. 2002; Duangmal et al. 2008). Anthocyanins possess antioxidative, antitumor, and anticarcinogenic activity (Fasoyiro et al. 2005; González-Molina et al. 2009). They are derivatives of the basic flavylium cation structure with electron-deficient nucleus which make them highly reactive and their reaction involves discoloration of the anthocyanin pigments. Factors like light, pH, temperature, oxygen, ascorbic acid, and sugar are contributing factors in degradation or stability of anthocyanins (Tsai and Huang 2004; Chumsri et al. 2008; Cisse et al. 2011).

Most people do not prefer beverages made from pure roselle as it has an acidic and bitter taste (Wong et al. 2002). Blending of the extract with juice from sweet tropical fruits such as mango can improve the aroma, taste and nutritional, and antioxidant properties of the juice blends. The choice of mango fruits in this study was due to an abundance seasonal availability, which normally led to high postharvest losses due to lack of sufficient market outlets, transport, storage facilities, and commercial fruit processing industries.

Packaging is an important aspect in the food processing industry as it serves the important functions of containing the food, protecting against chemical and physical damage whilst providing information on product features, nutritional status, and ingredient information (Anin et al. 2010). Various packaging materials such as high-density polyethylene (HDPE), polypropylene (PP), and glass are commonly used for packaging of fruit juice (Marsh and Bugusu 2007). Different packaging materials influence the quality of the stored products differently. Therefore, the study of the effect of packaging material on the quality parameters during storage is essential. In this study, roselle-mango juice blends were stored in plastic and glass bottles at ambient and refrigerated temperatures. The aim of this study was to determine effects of packaging materials, storage temperature, and time on physiochemical changes and antioxidant properties of roselle-mango juice blends.

## Materials and Methods

### Raw material and preparation of extract

Dark red dried roselle calyces were purchased from the Morogoro municipality market in Tanzania. Mango fruits (cv. “Dodo”) were purchased from the horticulture unit at Sokoine University of Agriculture, Tanzania.

Dried roselle calyxes (10% moisture content) were ground for 1 min using a blender (Kenwood BL 440, Boulogne, France). The ground calyces were mixed with water (1:10 w/v) and extracted using a water bath at 50°C for 30 min (Chumsri et al. 2008). The extract was filtered with a cheese cloth.

### Mango juice preparation

Fully matured and high-quality fruits of mango were used. Fresh fruits were thoroughly washed, peeled and cut into small pieces, and transferred to a juice extractor (Kenwood JE 810, Edinburgh, UK).

### Preparation of roselle-mango juice blends

Roselle-mango juice blends were formulated in the ratio of (80:20, 60:40, 40:60, and 20:80) roselle extract: mango juice pulp, “respectively.” Sodium benzoate (1 g/L) and citric acid (1 g/L) were added to all roselle-fruit blends as preservatives.

The juices were filled in 100 mL sterile plastic and glass bottles, loosely capped, and pasteurized in a water bath at a temperature of 82.5°C for 20 min and cooled rapidly to room temperature by immersing the bottles in cold water bath (Ndabikunze et al. 2010). The bottles were tightly capped, labeled and stored at 4°C and 28°C for 6 months. Samples were drawn for chemical analyses at 0, 2, 4, and 6 months of storage.

### Determination of pH, titratable acidity, and total soluble solids

The pH, titratable acidity (TA), and total soluble solids (TSS) of roselle-mango blends were determined according to AOAC (1995). The pH was measured using Hanna portable pH meter (Hanna, Cluj-Napoca, Romania). TA was determined titrimetrically using 0.1N sodium hydroxide and phenolphthalein as an indicator and was expressed as % malic acid, while TSS (^o^Brix) was measured with a hand refractometer (Mettler Toledo, Schwerzenbach, Switzerland) and expressed as %.

### Color measurements

The color for roselle-mango blends were measured using color chart (Natural Color system [NCS], Stockholm Sweden) followed by measuring the standard color with a Chroma Meter Minolta CR-400/410 (Minolta Co., Osaka, Japan) with the reflectance mode with D_65_ illuminant and 2° observer angle. Samples were measured against a white ceramic reference plate.

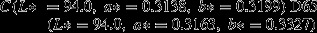
Color values were expressed as L* for lightness, a* for redness, and b* for yellowness.

### Reducing sugars

Reducing sugars (RS) were determined by the Luff–Schoorl method as described by Egan et al. (1981). Two grams of sample were weighed into a 100 mL Erlenmeyer cylinder and 90 mL distilled water, 5 mL Carrez I, and 5 mL Carrez II solution were added. The solution was mixed and filtered with Whatman filter (no. 542), and 10 mL of filtrate was transferred into a 250 mL Erlenmeyer flask, 10 mL of copper reagent was added, and then swirled. The solution was boiled in a direct flame for 3 min, cooled with tap water, and 1 g potassium iodide and 10 mL 6N HCl were added. This mixture was titrated with 0.1N Na_2_S_2_O_3_ until a yellow color appeared followed by adding a few drops of starch solution and titrated continuously until the blue color disappeared. Sugar content was then determined by interpolation in a table (Egan et al. 1981) after subtracting the blank assay to the volume of sodium thiosulfate of the titration. The results are expressed in mg/100 g fresh weight (FW).

### Determination of Vitamin C

Vitamin C content for the roselle-mango juice blends were determined according to the Folin–Ciocalteu reagent (FCR) method with modifications (Dashman et al. 1996), where 20 mL of sample was pipetted into a 100 mL volumetric flask followed by 2 mL of 10% tetrachloroacetic acid (TCA) solution and diluted to the 100 mL with distilled water. The sample was poured into a conical flask, swirled gently for 1 min, and left to stand for 1 min and filtered (Whatman filter no 542). One mL of the sample or 1 mL of standard solution (20 mg/100 mL) was pipetted into a test tube followed by 3 mL distilled water and 0.4 mL (1:10) Folin reagent. Mixing followed and thereafter the mixture was incubated at room temperature for 10 min. The absorbance was read at 760 nm using a Jenway 6405 UV/VIS Spectrophotometer (Jenway, Essex, UK). The results are expressed in mg/100 g FW.

### Determination of antioxidant activity

Antioxidant activity for the roselle-mango blends was determined by the ferric-reducing ability of plasma (FRAP) assay (Benzie and Strain 1996) with some modifications. Three milliliters of freshly prepared FRAP solution (0.3 mol/L acetate buffer (pH 3.6) containing 10 mmol/L 2,4,6-tripyridyl-s-triazine (TPTZ) in 40 mmol/L HCl and 20 mmol/L FeCl_3_·6H_2_O) and 100 *μ*L of sample (standard) was incubated at 37°C for 4 min, absorbance was measured at 593 nm using a spectrophotometer. An intense blue color was formed when the ferric-tripyridyltriazine (Fe^3+−^ TPTZ) complex reduced to the ferrous (Fe^2+^) form. A range of FeSO_4_·7H_2_O concentrations from 0.25 to 2.0 mmol/L was used to prepare the calibration curve. The results are expressed as millimoles of (Fe^2+^) per liter of FW (mmol (Fe^2+^)/L FW).

### Total phenolic content

Total phenolic content (TPC) for the roselle-mango blends was determined according to the Folin-Ciocalteu method with modifications (Singleton et al. 1999). An aliquot of 300 *μ*L sample solution was mixed with 1.5 mL of Folin-Ciocalteu's reagent (diluted 10 times), and 1.2 mL of sodium carbonate (7.5% w/v). After incubation at room temperature for 30 min in the dark, the absorbance was measured at 765 nm. Gallic acid (0–5 mmol/L/100 mL) was used for calibration of a standard curve. The results are expressed as milligrams of gallic acid equivalents per 100 g of FW (mg GAE/100 g FW).

### The total monomeric anthocyanin content

The total monomeric anthocyanin (TMA) content for roselle-mango blends was carried out using the pH differential method (Lee et al. 2005). Absorbance was measured at 520 and 700 nm using a spectrophotometer. The absorbance (*A*) of the sample was then calculated according to the following formula:

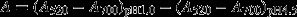


The monomeric anthocyanin pigment content in the original sample was calculated according to the following formula:

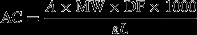
where *A* is the difference of sample absorbance between pH 1.0 and 4.5, *ε* is the molar extinction coefficient for cyanidin-3-glucoside (26,900 L/mol-cm), *L* is the path length of the spectrophotometer cell (1.0 cm), DL is the dilution factor and molecular weight (MW) of cyanidin-3-glucoside (449.2 g/mol), and 1000 is the factor for conversion from g to mg. The result are expressed as mg cyanidin-3-glucoside equivalent/L extract (mg cyn-3-glu/L) FW.

### Statistical analyses

All the tests were performed in triplicate and the results averaged (*n* = 3). Similar trends were observed in all the roselle-mango juice blends hence only one blend (40% roselle) was used in analysis of variance (ANOVA) using Minitab statistical software (Release 16.1 Minitab Inc., State College, PA). Multifactorial analysis of variance (General Linear Model [GML] was applied using a factorial design with three factors, including packaging bottles (plastic, glass), storage temperature (ambient A, refrigerated R), and storage time (0, 2, 4, 6 months). Principal component analysis (PCA) was used to evaluate seasonal variation of blends stored in plastic bottles (2011 and 2012 seasons), using Unscrambler X 10.2 (Camo Software AS, Oslo, Norway).

## Results and Discussion

The initial physiochemical properties of roselle-mango juice blends (2011 and 2012) are shown in Table1. The TSS and RS increased with increased concentration of mango juice in the blends as the fruit juice is known to contain high-sugar content while roselle extract is low in sugar content (Wong et al. 2002). The pH of the roselle-mango fruit juices was decreased with decreasing concentration of roselle extract as roselle is low in pH while TA was increased with increased concentration of roselle extract. TMA, total phenol and antioxidant activity (FRAP) also increased with increased concentration of roselle extract in the blends as the roselle extract is known to be a good source of anthocyanins (Wong et al. 2002).

**Table 1:** Initial physiochemical and antioxidant properties of roselle extract, mango juice, and roselle-mango juice blends (2011, 2012).

Year	Blends	TSS	pH	TA	RS	L^*^	a^*^	b^*^	Vit C	TMA	TPC	FRAP
2011	0R	14.0a ± 0.50	3.4a ± 0.12	0.3c ± 0.43	5.9a ± 0.05	42.4a ± 0.7	14.3e ± 0.3	43.9a±0.6	62.2a ± 0.00	48.0f ± 0.75	10.9e ± 5.22	1.28f ± 0.00
	20R	10.6b ± 0.49	2.8b ± 0.14	1.4b ± 0.00	5.6b ± 0.00	18.6b ± 0.4	16.4d ± 0.2	8.5b ± 0.2	58.5b ± 0.00	134.7e ± 1.50	21.3d ± 0.01	1.45e ± 0.00
	40R	9.9b ± 0.19	2.7b ± 0.01	1.9a ± 0.43	5.1c ± 0.00	17.6c ± 0.3	18.1c ± 0.7	7.7c ± 0.5	53.0c ± 0.00	282.6d ± 1.81	28.8c ± 0.03	1.58d ± 0.00
	60R	7.5c ± 0.63	2.4c ± 0.06	1.6b ± 0.40	4.5d ± 0.00	16.1d ± 0.2	19.2a ± 0.4	5.6d ± 0.2	44.4d ± 0.00	335.2c ± 1.54	37.9c ± 0.03	1.66c ± 0.00
	80R	6.9c ± 0.20	2.6c ± 0.01	1.4b ± 0.00	3.5e ± 0.00	14.7e ± 0.1	20.0a ± 0.5	4.7e ± 0.6	40.0e ± 0.00	493.5b ± 5.15	53.7b ± 0.02	1.80b ± 0.00
	100R	5.7d ± 0.10	2.3d ± 0.01	1.9a ± 0.00	2.4f ± 0.00	14.3e ± 0.00	20.6a ± 0.0	3.9f ± 0.0	37.4f ± 0.00	555.3a ± 2.03	54.6a ± 0.80	1.87a ± 0.01
2012	0R	15.5a ± 0.13	3.1a ± 0.01	0.3f ± 0.02	5.2a ± 0.02	42.4a ± 0.80	14.6e ± 0.02	44.5a ± 0.05	65.3a ± 0.01	32.9f ± 0.01	14.5f ± 0.19	1.42e ± 0.01
	20R	13.8b ± 0.07	2.9b ± 0.01	0.8e ± 0.02	4.4b ± 0.00	18.6b ± 0.03	16.7d ± 0.04	8.5b ± 0.06	60.5b ± 0.01	82.4e ± 0.05	23.4e ± 0.01	1.48d ± 0.02
	40R	11.2c ± 0.12	2.5c ± 0.01	1.3d ± 0.03	3.6c ± 0.01	17.9b ± 0.03	18.3c ± 0.49	7.8c ± 0.07	55.1c ± ± 0.02	236.3d ± 0.38	30.8d ± 0.01	1.58c ± 0.01
	60R	10.0d ± 0.13	2.4d ± 0.01	1.5c ± 0.03	2.9d ± 0.01	16.3c ± 0.36	19.8b ± 0.03	5.7d ± 0.06	47.2d ± 0.03	280.5c ± 0.01	38.4c ± 0.12	1.63b ± 0.00
	80R	8.0e ± 0.10	2.2e ± 0.01	1.7b ± 0.01	2.5e ± 0.00	14.9d ± 0.03	20.7a ± 0.02	4.7e ± 0.09	42.7e ± 0.05	464.2b ± 0.00	54.6b ± 0.12	1.86a ± 0.02
	100R	5.9f ± 0.07	2.1f ± 0.01	1.8a ± 0.03	2.0f ± 0.03	14.5e ± 0.02	20.9a ± 0.02	3.7f ± 0.04	39.3f ± 0.08	572.3a ± 0.01	56.3a ± 0.04	1.99a ± 0.04

100R, 100% roselle; 80R, 80% roselle; 60R, 60% roselle; 40R, 40% roselle; 20R, 20% roselle; 0, 0% roselle. TSS, total soluble solids (°brix); TA, titratable acidity (%); RS, reducing sugars (mg/100 g FW); Vit C, vitamin C (mg/100 g FW); FRAP, ferric-reducing ability of plasma (mmol/100 g FW); TMA, total monomeric anthocyanins (mg/L FW); TPC, total phenolic content (mg/100 g GAE FW); L^*^, lightness; a^*^, redness; b^*^, yellowness. Data in columns for each year with different superscript are significantly different using Tukey's pairwise comparison test (*P* < 0.05).

TSS for roselle-mango blends stored in glass and plastic bottles ranged from 8.0 to 13.7 °Brix (28°C) and 8.0–14.1 °Brix (4°C) while RS ranged from 2.5 to 5.7 mg/100 g (28°C), and 2.5–5.1 mg/100 g (4°C) during 6 months of storage (Table2). The results showed TSS and RS of roselle-mango blends increased during storage under both storage temperatures. The increase in TSS and RS was significant (*P* < 0.001) with storage temperature and storage time and packaging × storage temperature (Table5).

**Table 2:** Initial and final total soluble solids (TSS) and reducing sugar (RS) of roselle-mango juice blends stored in glass and plastic bottles for 6 months.

	TSS (%)					RS (mg/100 g FW)				
Packaging material		Glass		Plastic			Glass		Plastic	
Storage month	0	6		6		0	6		6	
Storage temperature	BS	28°C	4°C	28°C	4°C	BS	28°C	4°C	28°C	4°C
80R	8.0 ± 0.10	8.4 ± 0.12	8.5 ± 0.09	8.4 ± 0.10	8.5 ± 0.09	2.5 ± 0.01	3.7 ± 0.01	3.2 ± 0.01	3.7 ± 0.01	3.2 ± 0.01
60R	10.0 ± 0.15	11.9 ± 0.2	12.2 ± 0.42	11.9 ± 0.05	12.2 ± 0.42	2.9 ± 0.01	4.1 ± 0.01	4.0 ± 0.01	4.0 ± 0.01	4.0 ± 0.01
40R	11.2 ± 0.14	11.2 ± 0.17	11.5 ± 0.15	11.4 ± 0.04	11.5 ± 0.15	3.6 ± 0.01	4.9 ± 0.00	4.2 ± 0.01	4.9 ± 0.00	4.2 ± 0.01
20R	13.8 ± 0.08	13.7 ± 0.11	14.1 ± 0.08	13.7 ± 0.11	14.1 ± 0.08	4.4 ± 0.01	5.7 ± 0.01	5.1 ± 0.01	5.7 ± 0.01	5.1 ± 0.01

TSS, total soluble solids; RS, reducing sugar; M, months; 80R, 80% roselle; 60R, 60% roselle; 40R, 100% roselle; 20R, 20% roselle.

The TSS and RS increased gradually throughout storage, this might be due to hydrolysis of polysaccharides into monosaccharides and oligosaccharides (Bhardwaj and Pandey 2011). Similar trend of increased TSS with storage time was observed in mango-sea buckthorn blended juice stored for 90 days (Khan et al. 2012) and pomegranate kokum mango blends stored for 150 days (Waskar and Gaikwad 2004).

pH for roselle-mango blends stored in glass and plastic bottles ranged from 2.2 to 3.3 (28°C) and 2.2–3.5 (4°C) while TA ranged from 0.79% to 1.61% (28°C), and 0.8–1.61% (4°C) during 6 months of storage (Table3). Roselle extract is known to have low pH and addition of mango juice increased pH of the blends. The changes of TA were affected significantly by storage temperature (*P* < 0.05) and packaging × storage temperature interaction (*P* < 0.001) while pH was affected by storage temperature, storage time, and their interactions (*P* < 0.001).

**Table 3:** Initial and final pH and titratable acidity (TA) of roselle-mango juice blends stored in glass and plastic bottles at 28°C and 4°C.

	pH					TA (%)				
Packaging material		Glass		Plastic			Glass		Plastic	
Storage month	0	6		6		0	6		6	
Storage temperature	BS	28°C	4°C	28°C	4°C	BS	28°C	4°C	28°C	4°C
80R	2.2 ± 0.01	2.7 ± 0.01	3.0 ± 0.02	2.5 ± 0.01	2.6 ± 0.01	1.70 ± 0.02	1.57 ± 0.02	1.6 ± 0.09	1.58 ± 0.05	1.57 ± 0.02
60R	2.4 ± 0.01	2.8 ± 0.01	3.0 ± 0.03	2.6 ± 0.01	2.7 ± 0.01	1.50 ± 0.03	1.51 ± 0.02	1.5 ± 0.02	1.51 ± 0.02	1.52 ± 0.02
40R	2.5 ± 0.01	2.9 ± 0.01	3.0 ± 0.01	2.8 ± 0.01	2.9 ± 0.15	1.37 ± 0.01	1.3 ± 0.02	1.4 ± 0.02	1.34 ± 0.02	1.35 ± 0.02
20R	2.9 ± 0.02	3.1 ± 0.01	3.2 ± 0.01	3.1 ± 0.01	3.1 ± 0.01	0.83 ± 0.02	0.79 ± 0.02	0.8 ± 0.02	0.80 ± 0.02	0.79 ± 0.02

BS, before storage; TA, titratable acidity; M, months; 80R, 80% roselle; 60R, 60% roselle; 40R, 100% roselle; 20R, 20% roselle.

Lightness (L*) for roselle-mango blends stored in glass and plastic bottles ranged from 13.3 to 18.6 (28°C) and 13.5–18.6 (4°C) for 6 months of storage as shown on Table4. The L* value which is an indicator of lightness of color had shown to decrease with increased storage time. The decrease might be due to nonenzymatic browning reactions occurred to mango juice during storage. Falade et al. (2004) reported 47.4% and 36.8% decrease of L* values in sweetened Julie and Ogbomoso mango juices stored at 25°C. Martí et al. (2002) also reported a significant decrease in *L* value during storage of pomegranate juice for 150 days at 25°C.

**Table 4:** Initial and final color parameters of roselle-mango juice blends stored in glass and plastic bottles at 28°C and 4°C.

	Lightness (*L*)			Redness (*a*)			Yellowness (*b*)		
Storage temp	BS	28°C	4°C	BS	28°C	4°C	BS	28°C	4°C
Storage time	0	6	6	0	6	6	0	6	6
Glass									
80R	14.9 ± 0.03	14.4 ± 0.41	14.5 ± 0.04	20.6 ± 0.03	19.5 ± 0.03	19.8 ± 0.05	4.7 ± 0.09	3.3 ± 0.02	4.3 ± 0.05
60R	16.3 ± 0.40	15.4 ± 0.03	15.7 ± 0.02	19.8 ± 0.03	18.3 ± 0.04	18.7 ± 0.03	5.7 ± 0.06	4.4 ± 0.040	4.6 ± 0.04
40R	17.9 ± 0.04	16.4 ± 0.02	16.5 ± 0.06	18.3 ± 0.53	17.2 ± 0.06	17.4 ± 0.04	7.8 ± 0.07	6.6 ± 0.07	7.1 ± 0.02
20R	18.6 ± 0.03	17.3 ± 0.04	17.6 ± 0.05	16.7 ± 0.05	14.6 ± 0.06	15.4 ± 0.03	8.5 ± 0.07	7.3 ± 0.05	7.5 ± 0.05
Plastic									
80R	14.9 ± 0.03	13.3 ± 0.03	13.5 ± 0.05	20.6 ± 0.03	17.8 ± 0.06	17.9 ± 0.07	4.7 ± 0.09	2.8 ± 0.06	2.7 ± 0.06
60R	16.3 ± 0.40	13.8 ± 0.05	14.2 ± 0.05	19.8 ± 0.03	17.1 ± 0.05	17.2 ± 0.04	5.7 ± 0.06	3.3 ± 0.05	3.7 ± 0.05
40R	17.9 ± 0.04	14.7 ± 0.06	15.1 ± 0.04	18.3 ± 0.53	15.3 ± 0.07	15.2 ± 0.05	7.8 ± 0.07	4.6 ± 0.41	5.9 ± 0.05
20R	18.6 ± 0.03	16.5 ± 0.08	16.8 ± 0.05	16.7 ± 0.05	16.1 ± 0.02	16.7 ± 0.04	8.5 ± 0.07	6.4 ± 0.08	6.4 ± 0.05

BS, before storage; 80R, 80% roselle; 60R, 60% roselle; 40R, 100% roselle; 20R, 20% roselle.

Redness (a*) for roselle-mango blends stored in glass and plastic bottles ranged from 14.6 to 20.6 (28°C) and 15.2–20.6 (4°C) for 6 months of storage. Yellowness (b*) for roselle-mango blends stored in glass bottles ranged from 2.7 to 8.2 (28°C) and 2.8–8.2 (4°C) after 6 months of storage. The yellowness in roselle-mango juice blends is due to the presence of carotenoids in mango juice. However, these carotenoids are highly susceptible to degradation by heat, low pH, and light exposure (Hewavitharana et al. 2013). The effect of packaging material, storage time, storage temperature, and their interactions, significantly (*P* < 0.001) affected the yellowness b* for roselle-mango juice blends (Table5).

**Table 5:** Effects of packaging, storage time and temperature on the quality of roselle-mango juice blends (40% roselle).

Source of variation	TSS	pH	TA	RS	TMA	Vit. C	TPC	FRAP	L^*^	a^*^	b^*^
Packaging (*A*)	ns	ns	ns	ns	<0.001	<0.001	<0.001	<0.001	<0.001	<0.001	<0.001
Temperature (*B*)	<0.001	<0.001	0.037	<0.001	<0.001	0.006	<0.001	ns	0.05	ns	<0.001
Time (*C*)	<0.001	<0.001	ns	<0.001	<0.001	<0.001	<0.001	<0.001	<0.001	<0.001	<0.001
*A* × *B*	<0.001	<0.001	<0.001	<0.001	<0.001	<0.001	<0.001	<0.001	ns	ns	<0.001
*A* × *C*	ns	<0.001	ns	ns	<0.001	<0.001	<0.001	<0.001	<0.001	<0.001	<0.001
*B* × *C*	ns	ns	ns	ns	<0.001	<0.001	<0.001	<0.001	<0.001	<0.001	<0.001

ns, not significant; TSS, total soluble solids; TA, titratable acidity; RS, reducing sugars; Vit C, vitamin C; FRAP, ferric-reducing ability of plasma; TMA, total monomeric anthocyanins; TPC, total phenolic content; L^*^, lightness; a^*^, redness; b^*^, yellowness.

**Figure 1:**
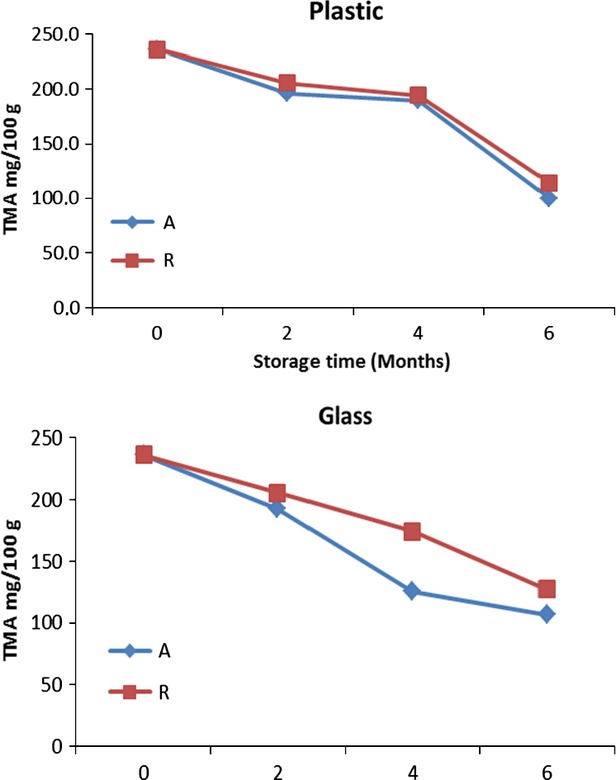
Total monomeric anthocyanin (TMA) for roselle-mango juice blend (40% roselle) stored in glass and plastic bottles at ambient (A) and refrigerated (R).

TMA of the roselle-mango juice blends (40%R) stored in glass bottles at 4°C were higher than those stored at 28°C (Fig.1). The decrease was significant (*P* < 0.05) during storage, irrespective of storage temperature and packaging material (Table5). Waskar and Gaikwad (2004) observed similar trends on pomegranate kokum mango- based blends stored for 150 days. The amount of anthocyanin remained after 6 months was (127.7–144.1 mg/100 g) at 4°C and 100–107 mg/100 g at 28°C in all roselle-mango blends (40%) stored in glass and plastic bottles, these amounts were sufficient to provide the amount of anthocyanins per day recommended by the United States of America and Finland (82 and 12.5 mg per day) by Wu et al. (2006).

**Figure 2:**
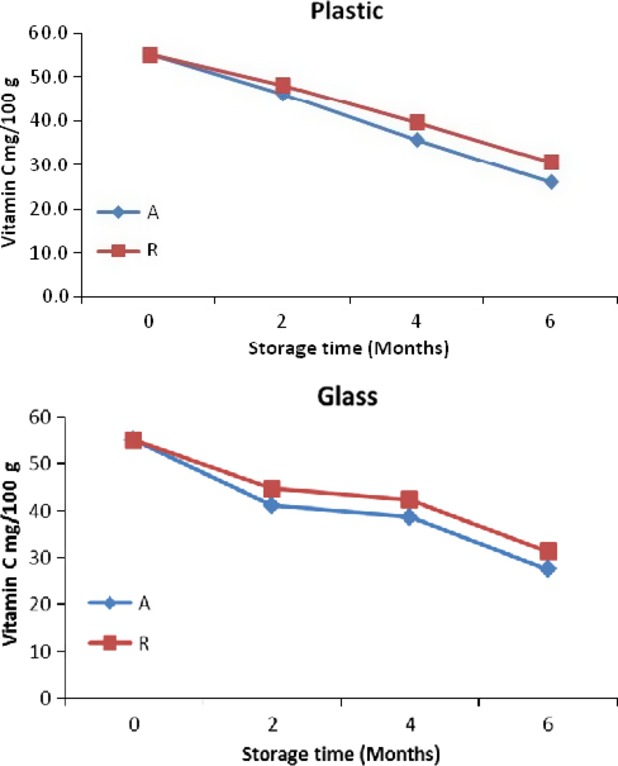
Vitamin C content for roselle-mango juice blend (40% roselle) stored in glass and plastic bottles at ambient (A) and refrigerated (R).

Vitamin C content of the blends decreased significantly (*P* < 0.05) with increased storage period because vitamin C can easily be oxidized in the presence of oxygen by both enzymatic and nonenzymatic catalyst (Jawaheer et al. 2003).Vitamin C losses was lower in roselle-mango juice blends stored in glass bottles (Fig.2). Similar results were observed by Alaka et al. 2003 when mango juices were packaged in polyethylene films, polyethylene tetraphthalate (PET or plastic) bottles and transparent glass bottles, and stored at 6°C, 26°C, and 34°C. Despite the fact that the vitamin C losses in roselle-mango blends (40% roselle) stored at 4°C for 2 months was more than 45 mg per 100 mL, i.e., only 100 mL of the blends will contain sufficient vitamin C to provide the recommended daily allowance (RDA) for adults, which is 45 mg (FAO/WHO 2001).

**Figure 3:**
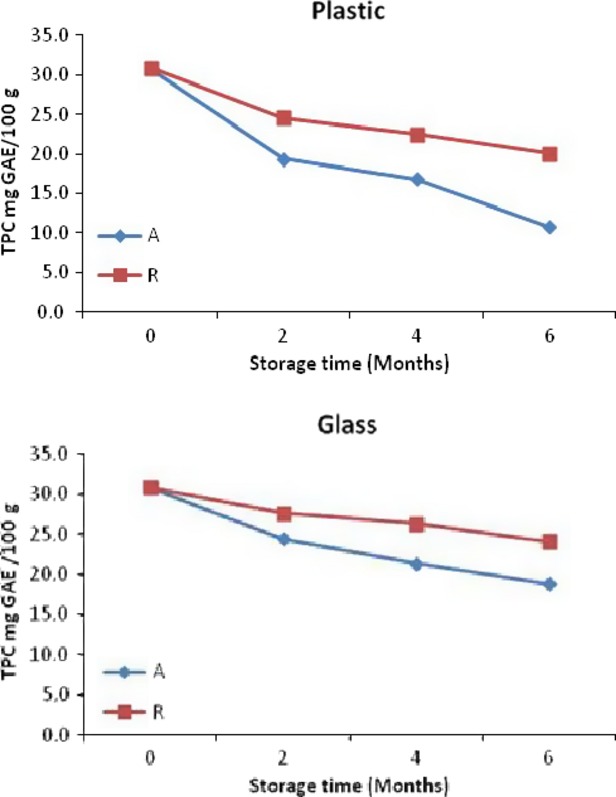
Total phenolic content (TPC) for roselle-mango juice blend(40% roselle) stored in glass and plastic bottles at ambient (A) and refrigerated (R).

Polyphenols are the most abundant antioxidants in the diet and are widespread constituents of fruits and vegetables (Fang et al. 2006). However, they are susceptible to degradation during storage, which was demonstrated by the value of 30.9 mg GAE/100 g initially for roselle-mango blends (40%R) which decreased to 18.8 (28°C) and 20.1 (4°C) after 6 months of storage (Fig.3). All variables (time, temperature, and packaging and interaction term, time × temperature, time × packaging, and temperature × packaging) significantly contributed to the loss of total phenol content (TPC) see Table5.

**Figure 4:**
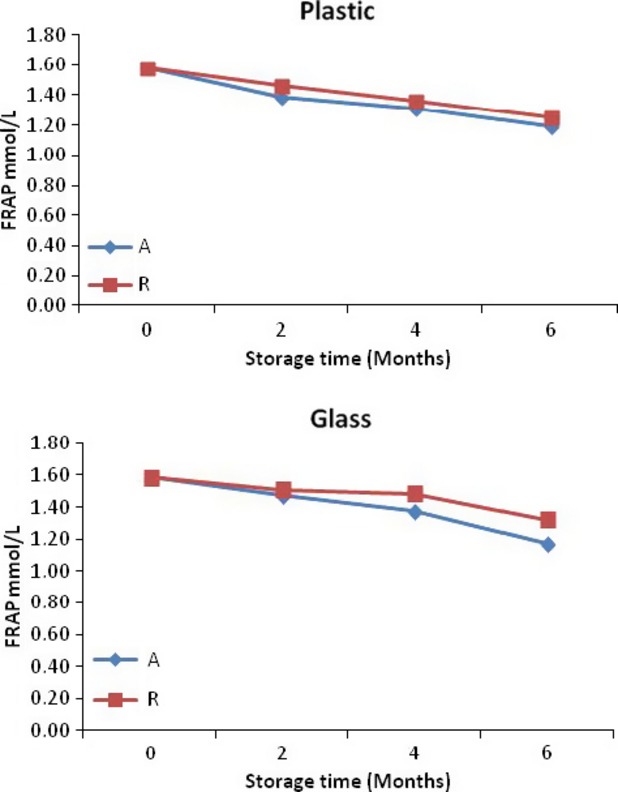
Antioxidant activity (FRAP) for roselle-mango juice blend (40% roselle) stored in glass and plastic bottles at ambient (A) and refrigerated (R).

FRAP for roselle-mango blends stored in glass and plastic bottles ranged from 1.04 to 1.86 mmol/L (28°C) and 1.19–1.86 mmol/L (4°C) after 6 months of storage (Fig.4). Despite marked losses of TMA in all the roselle-mango blends, FRAP value losses were less than 30% during storage, suggesting that polymeric compounds formed during storage might have compensated the loss of antioxidant activity due to degradation of monomeric anthocyanins (Tsai and Huang 2004).

**Figure 5:**
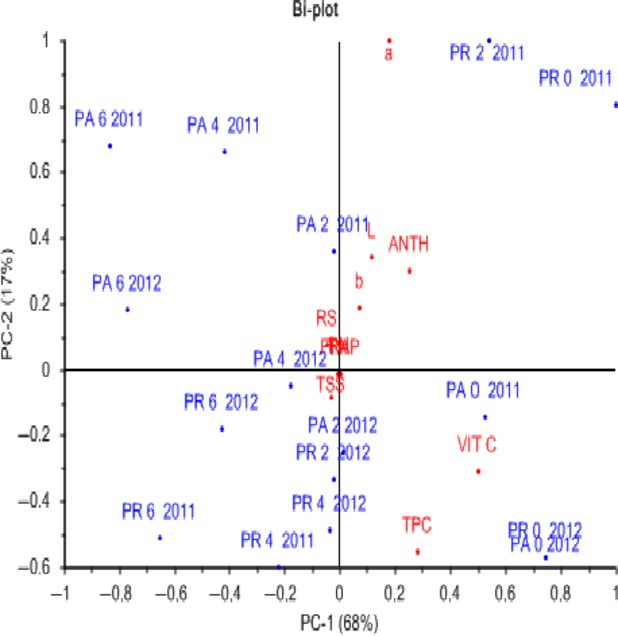
Bi-plot (scores and loadings) for roselle-mango juice blend stored at ambient and refrigerated temperature for 6 months (season 2011 and 2012).

In the case of seasonal variation and storage time and temperature, a bi-plot of observations and variables is shown in Figure5. Most of the variation (85%) was explained by the first two principle components (PC) with the first component (PC1) accounting for 68% and associated with parameters (TSS, Vitamin C, and TPC) and the second components account for 17% of the total variation associated with parameters (color L*, a*, b* RS, FRAP, and TMA). The PC1 explained roselle-mango juice blends stored in plastic bottles at refrigerated temperature with more blends from season 2012 while PC2 explained blends stored at ambient temperature with more blends from season 2011.

The roselle-mango juice blends stored at refrigerated temperature for zero and 2 months were on the positive side of PC2 while those blends stored for 4 and 6 months were on the negative side of PC2. Those blends stored at ambient temperature for 0 months were on the positive side of PC1 while those blends stored at ambient temperature for 2, 4, and 6 months were on the negative side of the PC1.

The results showed that most of 2012 blends had high levels of TPC, TSS, and Vitamin C (Fig.5 and Table1). Regardless of season or storage temperature, results on the bi-plots showed the effects of storage time on the blends as storage was progressing with storage at 4 and 6 months being on the negative side of the PCs. The Bi plots also showed the TPC, TMA, and Vitamin C were parameters mostly affected by the storage time regardless of storage temperature.

## Conclusions

The roselle-mango blends presented some chemical changes during 6 months storage. The most affected components were TMA, total phenols, and vitamin C. The blends stored at 28°C showed remarkable losses of TMA, TPC, and vitamin C as compared to 4°C, hence storage at 28°C should be avoided if good long-term preservation of the roselle-mango juice blends is desired due to retention of more TMA, TPC, and vitamin C. Packaging in glass bottles and storage at 4°C should be encouraged as it retains more vitamin C and TMA essential in antioxidant capacity of fruits and fruit products. Seasonality and packaging material, storage time, and temperature have shown to affect total monomeric anthocyanin contents, total phenol, and vitamin C content of the roselle-mango juice blends. The quantity of total monomeric anthocyanin and vitamin C remaining after 6 months of storage of the roselle-mango juice blends (40%R) was sufficient to provide recommended amount for daily intake of anthocyanins and vitamin C for adults.

## Conflict of Interest

None declared.
